# Postoperative Adjuvant Chemotherapy Regimen of CAPOX Combined With Ninjin'yoeito in an Elderly Patient With Stage III Colon Cancer: A Case Report

**DOI:** 10.3389/fnut.2020.00057

**Published:** 2020-04-30

**Authors:** Naoki Aomatsu, Yasutake Uchima, Gen Tsujio, Yoshinari Miyamoto, Takuma Okada, Shigeaki Kurihara, Shinji Matsutani, Toshiki Hirakawa, Takehiko Iwauchi, Junya Morimoto, Shigehito Yamagata, Kazunori Nakazawa, Takafumi Nishii, Akiko Tachimori, Kiyoshi Maeda, Katsumi Ikeda, Kazuhiro Takeuchi

**Affiliations:** ^1^Department of Gastroenterological Surgery, Osaka City General Hospital, Osaka, Japan; ^2^Department of Surgery Center, Fuchu Hospital, Osaka, Japan; ^3^Department of Breast Surgical Oncology, Osaka City General Hospital, Osaka, Japan

**Keywords:** CAPOX, ninjin'yoeito, colon cancer, adjuvant chemotherapy, kampo medicine

## Abstract

We report the successful management of stage III colon cancer in an elderly patient who received an adjuvant chemotherapy regimen of capecitabine plus oxaliplatin (CAPOX) with the Japanese kampo medicine ninjin'yoeito (NYT). A 75-year-old woman with a medical history of hypertension presented at another institution with fecal occult blood, and a colonoscopy that showed a type II tumor in the sigmoid colon. She was referred to our hospital for tumor resection, where colonoscopy confirmed the location of the type II tumor in the sigmoid colon. Histopathology of the biopsy specimen indicated a moderately differentiated tubular adenocarcinoma. Enhanced computed tomography of the thorax and abdomen indicated thickening of the sigmoid colon wall. Regional lymph node metastasis was suspected, but distant metastasis was not indicated. A blood examination revealed an elevated carcinoembryonic antigen (CEA) concentration (32.7 ng/ml). Following a diagnosis of cancer of the sigmoid colon, clinical stage IIIb [cT4a, N1b, M0], a laparoscopic sigmoid colectomy was performed without complications. The postoperative histopathological examination revealed a moderately differentiated to mucinous adenocarcinoma. Three of 16 retrieved lymph nodes contained malignant cells. The final tumor classification was Stage IIIb [pT4a, pN1b, M0]. The patient recovered uneventfully, and was discharged 10 days after surgery with a recommendation for adjuvant chemotherapy with CAPOX starting 4 weeks after surgery. The patient also received 7.5 g of NYT daily throughout the adjuvant chemotherapy course. She did not report any loss of appetite, general fatigue, peripheral neuropathy, neutropenia, or febrile neutropenia. During a 1-year postoperative follow-up, she has not experienced any recurrence. We conclude that NYT might be useful for reducing the adverse effects of anticancer therapy, particularly in elderly patients.

## Introduction

Combinations of oxaliplatin (L-OHP) with fluoropyrimidine drugs have been used as standard adjuvant chemotherapy regimens for the treatment of stage III colon cancer since 2004. Although elderly patients reap the same benefits from these regimens as younger patients, the main adverse effect of L-OHP is peripheral neuropathy. In previous reports, ninjin'yoeito (NYT) was reported to be useful for reducing adverse effects such as anticancer anemia, peripheral neuropathy, and cancer cachexia (1–4). We report a case of postoperative adjuvant chemotherapy comprising capecitabine plus L-OHP (CAPOX) combined with NYT for the treatment of stage III colon cancer in an elderly patient.

## Case Presentation

A 75-year-old woman with a medical history of hypertension presented at another institution with fecal occult blood, and a colonoscopy showed a type II tumor in the sigmoid colon. She was referred to our hospital for tumor resection, where colonoscopy determined that the tumor was located 23 cm from the anal verge. Histopathology of a biopsy specimen revealed a moderately differentiated tubular adenocarcinoma. Enhanced computed tomography of the thorax and abdomen showed sigmoid colon wall thickening. Regional lymph node metastasis was suspected, but no evidence of distant metastasis was observed. A blood examination revealed an elevated carcinoembryonic antigen (CEA) concentration (32.7 ng/ml). Following a diagnosis of cancer of the sigmoid colon, clinical stage IIIb [cT4a, N1b, M0], a laparoscopic sigmoid colectomy was performed without complications. The postoperative histopathological examination revealed a moderately differentiated to mucinous adenocarcinoma. Three of the 16 retrieved lymph nodes contained malignant cells. Finally, the cancer was classified as stage IIIb [pT4a, pN1b, M0]. The patient recovered uneventfully and was discharged 10 days after the surgery. Following the diagnosis of stage III colorectal cancer, the patient was recommended to receive adjuvant chemotherapy with CAPOX starting 4 weeks after surgery. The selected regimen consisted of capecitabine (1,000 mg/m^2^ orally twice daily) for 14 days and L-OHP (130 mg/m^2^ intravenous infusion) on the first day of each cycle, with a periodicity of 3 weeks over 3 months (four cycles). The anticancer drug dosage was reduced to 80% because of the patient's age. The patient had postoperative physical weakness and appetite loss, and also received 7.5 g of NYT daily throughout the course of adjuvant chemotherapy. She did not report any events of peripheral loss of appetite, general fatigue, peripheral neuropathy, neutropenia, or febrile neutropenia. Adverse effects were graded according to the Common Terminology Criteria for Adverse Events (CTCAE) v5.0. She has not experienced any recurrence during a 1-year postoperative follow-up.

## Discussion

Herein, we report the successful management of stage III colon cancer in an elderly patient via adjuvant CAPOX chemotherapy combined with NYT. Adjuvant chemotherapy improves overall survival in patients with resected stage III colorectal cancer (5). The MOSAIC Multicenter International Study (6) revealed that a regimen of FOLFOX [fluorouracil (FU) bolus and continuous infusion combined with leucovorin (LV) and L-OHP] significantly improved 3-year disease-free survival outcomes when compared with fluorouracil and leucovorin alone. Additionally, CAPOX improved overall survival outcomes in patients with resected stage III colon cancer when compared with bolus FU after a median follow-up of almost 7 years (7). CAPOX allows for central venous (CV)-port-free administration. Therefore, this regimen might be considered a standard adjuvant treatment option for patients with stage III colon cancer.

Elderly patients receive the same survival benefit from these regimens as younger patients. In both older (≥70 years) and younger patients, adjuvant 5-FU therapy has been significantly associated with reduced mortality in randomized controlled trials (8). Haller reported that in a pooled analysis of individual patient data from four randomized controlled trials of CAPOX/FOLFOX vs. LV/5-FU [NSABP C-08 (9), XELOXA (7), X-ACT (10), and AVANT (11)], disease-free survival benefits were observed regardless of age or medical comorbidity. However, the benefits were modestly attenuated in patients aged ≥70 years, with a hazard ratio of 0.77 (*P* < 0.014) vs. 0.68 (*P* < 0.0001) among patients aged <70 years (12).

Peripheral neuropathy is the main adverse effect of L-OHP therapy. In the MOSAIC study, grade 2 and 3 peripheral sensory neuropathy was observed during treatment in 31.4 and 12.5% patients in the FOLFOX group (13). During treatment with L-OHP chemotherapy, patients have experienced acute and chronic mechanical hyperalgesia and cold allodynia. The dose and duration of therapy are limited once a patient develops peripheral neuropathy, leading to a reduced quality of life. The cumulative administered dose of L-OHP increases the risk of associated sensory neurotoxicity (13, 14). In the MOSAIC trial (13), the frequency of grade 3 peripheral sensory neuropathy among patients receiving L-OHP persisted over time (1.3% at 12 months and 0.7% at 48 months after treatment). This toxic adverse effect may be severe and can persist long after treatment is completed, leading to potentially life-long effects on the patients' activities of daily living (15).

To date, various integrative approaches, including Japanese kampo medicine, have been used in an attempt to prevent the adverse effects of chemotherapy. Kampo medicines are currently used to treat several types of diseases, and are also used to improve the quality of life of patients throughout the world and especially in Asian countries (3). Of the traditional medicine components prescribed to prevent L-OHP-induced peripheral neuropathy, goshajinkigan has been the most popular with animal experiments (16–18). However, goshajinkigan could not prevent L-OHP-induced peripheral neurotoxicity in colorectal cancer patients treated with FOLFOX regimens in a randomized phase III clinical trial (19).

NYT is a Japanese kampo medicine composed of 12 herbal plants that is used to facilitate disease recovery and improve several symptoms, such as anemia, anorexia, and fatigue. In previous reports, NYT appeared to be useful for reducing the adverse effects of anticancer anemia, peripheral neuropathy and cancer cachexia (1–4). NYT should be administered at the same time as adjuvant chemotherapy. At that time, the indicating disease, such as postoperative physical weakness, general fatigue, appetite loss, and anemia, must be present. Some references indicate that more human data, both younger and elderly patients, is needed to validate the efficacy of NYT in managing adverse effects of a systemic chemotherapy regimen (20, 21). In mice, NYT improved 5-FU-induced anemia and increased the populations of burst-forming unit-erythroid cells and colony-forming unit-erythroid cells in bone marrow (22).

In an *in vitro* study, Suzuki reported that an extract of NYT prevented L-OHP-induced neurodegeneration in PC12 cells (1). Particularly, ginseng extract appeared to exert the strongest protective effect against neurodegeneration among the 12 herbal components of NYT. In a mouse model experiment, NYT and ginseng reduced L-OHP-induced neurite damage and neuropathic pain. In an *in vitro* study, L-OHP treatment suppressed neurite outgrowths from primary dorsal root ganglion cells. NYT extract blocked this suppression in a concentration-dependent manner. Ginseng showed a protective effect against neurite damage induced by L-OHP, and one of its active ingredients was identified as ginsenoside Rg3 (4). In addition to NYT, other kampo medicines, such as ginseng and hochuekkito also reduce the adverse effects of anticancer therapy. Hochuekkito is not expected to improve peripheral neuropathy, but has been noted to improve gastrointestinal conditions and increase physical strength, and is expected to improve immune function (23). Consistent with those earlier observations, the administration of NYT reduced the adverse effects associated with adjuvant CAPOX chemotherapy, such as peripheral neuropathy, neutropenia, and febrile neutropenia in our elderly patient with stage III colorectal cancer.

In conclusion, our observations suggest that NYT might be useful for reducing the adverse effects of anticancer therapy in elderly patients.

## Data Availability Statement

The raw data supporting the conclusions of this article will be made available by the authors, without undue reservation, to any qualified researcher.

## Ethics Statement

The studies involving human participants were reviewed and approved by Ethical Committee of the Fuchu Hospital. The patients/participants provided their written informed consent to participate in this study.

## Author Contributions

NA made a substantial contribution to the study conception, conducted a literature search, and drafted the manuscript. NA and GT contributed to the acquisition of data. NA, SK, TO, and YU performed the surgery. NA, YU, GT, YM, TO, SK, SM, TH, TI, JM, SY, KN, TN, AT, KM, KI, and KT reviewed the manuscript and gave final approval for publication. All authors read and approved the final manuscript.

## Conflict of Interest

The authors declare that the research was conducted in the absence of any commercial or financial relationships that could be construed as a potential conflict of interest.
